# RBM14 Modulates Tubulin Acetylation and Regulates Spindle Morphology During Meiotic Maturation in Mouse Oocytes

**DOI:** 10.3389/fcell.2021.635728

**Published:** 2021-02-02

**Authors:** Hao Qin, Yi Qu, Yi-Feng Yuan, Yang-Yang Li, Jie Qiao

**Affiliations:** ^1^Center for Reproductive Medicine, Department of Obstetrics and Gynecology, Peking University Third Hospital, Beijing, China; ^2^National Clinical Research Center for Obstetrics and Gynecology (Peking University Third Hospital), Beijing, China; ^3^Key Laboratory of Assisted Reproduction (Peking University), Ministry of Education, Peking University, Beijing, China; ^4^Beijing Key Laboratory of Reproductive Endocrinology and Assisted Reproductive Technology, Beijing, China; ^5^Research Units of Comprehensive Diagnosis and Treatment of Oocyte Maturation Arrest, Chinese Academy of Medical Sciences, Beijing, China

**Keywords:** RBM14, oocyte, meiosis, spindle, acetylation

## Abstract

RBM14 is an RNA-binding protein that regulates spindle integrity in mitosis; however, its functions during meiosis are still unclear. In this study, we discovered that RBM14 expression was down-regulated in oocytes from old mice. The RBM14 distribution at different stages of meiosis was explored, while it presents overlapped localization patterns with α-tubulin in MI- and MII-stage oocytes. Treatment of MI-stage oocytes with spindle-perturbing agents revealed that RBM14 was co-localized with microtubules. RBM14 knockdown with RBM14-specific morpholino showed that RBM14-depleted oocytes underwent symmetric division compared to the controls. RBM14 knockdown also resulted in spindle defects and chromosome abnormalities during oocyte maturation, presumably due to α-tubulin hyperacetylation. Co-immunoprecipitation analysis demonstrated that RBM14 is interacted with endogenous α-tubulin in mammalian cells. These findings indicate that RBM14 is an essential modulator of oocyte meiotic maturation by regulating α-tubulin acetylation to affect spindle morphology and chromosome alignment. Consequently, RBM14 represents a potential biomarker of oocyte quality and a novel therapeutic target in women with oocyte maturation failure.

## Introduction

In recent decades, there has been a trend toward the postponement of first birth due to a diverse array of influences, including women's rising education, participation in the labor force, individualization and value changes (Mills et al., 2011). Accordingly, the age of women at the birth of their first child has been increasing (Mathews and Hamilton, 2016). Reproductive aging in females results in a progressive decline in both the quantity and the quality of oocytes, which are risk factors for infertility, aneuploidy, and low pregnancy rates (Broekmans et al., 2009). The emergence of assisted reproductive technology (ART) offers hope to couples suffering from infertility, and the proportion of women of advanced maternal age who are seeking ART interventions is increasing (Centers for Disease Control Prevention, 2009, 2019; Shea et al., 2015). Thus, there is an unmet clinical need to improve oocyte quality and explore the specific etiopathological mechanisms of oocyte maturation failure to ensure better clinical outcomes following ART procedures.

In mammals, oocytes within ovarian follicles are characterized by the presence of the germinal vesicle (GV). A surge of luteinizing hormone triggers the resumption of meiosis and induces germinal vesicle breakdown (GVBD). Subsequently, oocytes progress to metaphase I (MI) with spindle formation, and chromosomes align at the spindle equator. Thereafter, oocytes arrest at metaphase II (MII), with chromosome segregation and extrusion of the first polar body, until fertilization (Sanders and Jones, 2018; Pan and Li, 2019). Accurate progression through meiosis is a crucial event involving post-translational modifications of various proteins. Among the molecular mechanisms controlling oocyte meiotic maturation, tubulin acetylation is essential for microtubule structure and organization (Amargant et al., 2019). Previous studies in mice showed that female aging resulted in hyperacetylation of α-tubulin and assembly failure of the meiotic apparatus in oocytes (He et al., 2019).

In humans, RNA-binding proteins (RBPs) account for an estimated 7.5% of the coding genome (Weiße et al., 2020). RBPs mediate post-transcriptional gene regulation by controlling processes such as RNA splicing (Fu and Ares, 2014), stability (Hasan et al., 2014), translation (Abaza and Gebauer, 2008), and localization (Shav-Tal and Singer, 2005). Most RBPs contain one or more RNA recognition motifs (RRMs), which are crucial for sequence-specific recognition of RNA (Maris et al., 2005). RNA-binding motif protein 14 (RBM14) harbors two N-terminal RRMs and one C-terminal disordered prion-like domain that functions as an RNA splicing modulator and transcription co-activator (Iwasaki et al., 2001). Recent reports show that RBM14 interacts with a novel long intergenic non-coding RNA in the regulation of adipocyte differentiation (Firmin et al., 2017) and RBM14 participates in the repair of double-strand breaks via the canonical nonhomologous end joining pathway (Jang et al., 2020). During mitosis, RBM14 depletion affects spindle integrity and chromosome segregation by inducing ectopic assembly of centriolar protein complexes (Shiratsuchi et al., 2015).

Previously, we have provided a framework for understanding the transcriptome of human folliculogenesis and early embryos using single-cell RNA-seq analysis (Yan et al., 2013; Zhang et al., 2018). Our transcriptome data showed that RBM14 was detected at different stages during oocyte maturation and preimplantation embryonic development. However, the functions of RBM14 during oocyte meiotic maturation in mammals remain to be elucidated. In the current study, we used loss-of-function and co-immunoprecipitation (Co-IP) experiments to show that RBM14 is a potential factor contributing to oocyte quality and meiotic maturation of oocytes.

## Materials and Methods

### Oocyte Collection and Culture

Approval for all animal experiments was provided by the Institutional Animal Care and Use Committee of Peking University. Animal experiments were performed according to the National Institutes of Health Guidelines for Use of Laboratory Animals. Female Institute of Cancer Research (ICR) mice (aged 6–8 weeks or 42–45 weeks) were provided by Beijing Vital River Laboratory Animal Technology Co., Ltd. (Beijing, China). Mice were housed in standard cages under a 12:12 h light-dark cycle at 20–23°C with unlimited access to water and food. Fully grown intact GV-stage oocytes were collected from ovaries of mice that had been euthanized by cervical dislocation 44–46 hours (h) after intraperitoneal injection of 5 IU pregnant mare serum gonadotropin (PMSG). Fully grown cumulus-enclosed oocytes were collected by rupturing antral ovarian follicles. Cumulus cells were removed by gentle washing, and denuded oocytes were transferred to pre-warmed M2 medium (Sigma; M7167) supplemented with 2.5 μM milrinone to arrest them at the GV stage. *In vitro* maturation was achieved by culturing oocytes in M16 medium (Sigma; M7292) under mineral oil at 37°C in a 5% CO_2_ incubator.

### Western Blot Analysis

Mouse oocytes were lysed in Laemmli sample buffer containing protease inhibitor and heated at 100°C for 10 min. Total oocyte proteins were subjected to 10% SDS-PAGE and transferred to methanol-treated polyvinylidene fluoride (PVDF) membranes (Millipore; IPVH00010). Membranes were blocked in 5% non-fat milk/TBST for 1 h at room temperature (RT) and incubated with rabbit polyclonal anti-RBM14 antibody (Sigma; HPA006628; 1:500), mouse monoclonal anti-acetyl-tubulin (Lys-40) antibody (Sigma; T7451; 1:1,000), rabbit polyclonal anti-α-tubulin antibody (Proteintech; 11224-1-AP; 1:1,000), or mouse monoclonal anti-β-actin antibody (Sigma; A5441; 1:1,000) overnight at 4°C. Membranes were washed three times for 10 min each in TBST and incubated with HRP-conjugated goat anti-rabbit IgG (H + L) (Proteintech; SA00001-2; 1:3,000), HRP-conjugated goat anti-mouse IgG (H + L) (Proteintech; SA00001-1; 1:3,000), or HRP-conjugated mouse anti-rabbit (light-chain specific) (CST; 93702; 1:3,000) secondary antibodies for 1 h at RT. Protein bands were visualized with ECL Plus Western Blotting Detection System (Tanon-5200).

### Control or RBM14 Morpholino (MO) Microinjection

Endogenous RBM14 proteins were knocked down in mouse GV-stage oocytes using a microinjection system (Eppendorf, Hamburg, Germany). RBM14 MO 5′-AAA TCT TCA TTT TGC CGC CGC AAC C-3′ (Gene Tools, Philomath, OR, USA) was diluted with water to provide a 1 mM stock solution, and then ~5–10 pl of RBM14 MO was injected into each GV-stage oocyte. Nontargeting MO 5′-CCT CTT ACC TCA GTT ACA ATT TAT A-3′ served as the negative control. Meiosis was resumed in the fresh M16 medium after culturing oocytes in M2 medium supplemented with 2.5 μM milrinone for 20 h.

### Immunofluorescence Staining

Oocytes were fixed in 4% paraformaldehyde/PBS (pH 7.4) for 30 min at RT, permeabilized with 0.5% Triton X-100/PBS for 20 min, blocked in 1% BSA/PBS for 1 h, and incubated with primary antibodies overnight at 4°C. Goat anti-rabbit IgG (Proteintech; B900610) served as the negative control. Oocytes were washed three times in PBS containing 0.1% Tween 20 and 0.01% Triton X-100 and incubated with fluorescent secondary antibodies (Alexa Fluor 488 donkey anti-mouse IgG [H + L] [Invitrogen; A21202] or Alexa Fluor 555 goat anti-rabbit IgG [H + L] [Invitrogen; A21428]) at RT for 2 h in the dark. Oocytes were stained with Hoechst 33342 for 10 min to visualize chromosomes and then observed with a confocal laser scanning microscope (Carl Zeiss 710, Germany).

### Immunoprecipitation (IP)

IP analysis was performed with the Pierce Crosslink IP Kit (Thermo Fisher Scientific; 26147), according to the manufacturer's instructions. Briefly, a rabbit polyclonal anti-RBM14 antibody (Abcam; ab70636) or anti-α-tubulin antibody (Proteintech; 11224-1-AP) was conjugated to pierce protein A/G plus agarose with disuccinimidyl suberate (DSS) crosslinking. Mouse NIH/3T3 whole cell lysate was harvested in IP lysis buffer (0.025 M Tris, 0.15 M NaCl, 0.001 M EDTA, 1% NP-40, 5% glycerol, pH 7.4) containing EDTA-free protease inhibitor cocktail (Roche; 4693132001) and pre-cleared with pierce control agarose resin. The input sample comprised 10% of the lysate. The anti-RBM14 or anti-α-tubulin antibody-crosslinked resin was incubated with pre-cleared lysate overnight at 4°C on a rotator and then washed three times in IP wash buffer. The IP products were eluted by elution buffer and neutralizing with 1 M Tris-HCl buffer (pH 9.5). The eluates were separated by SDS-PAGE and transferred onto PVDF membranes. IP with nonspecific rabbit IgG antibody (Proteintech; B900610) served as the negative control.

### Statistical Analysis

Statistical analysis was performed with SPSS software (SPSS Inc, Chicago, IL). Data were evaluated with the Student's *t*-test and presented as mean ± SEM from three independent experiments. *P* < 0.05 was considered statistically significant.

## Results

### RBM14 Level Was Decreased in Oocytes From Aged Mice and Increased Progressively During Oocyte Meiotic Maturation

**Figure 1:**
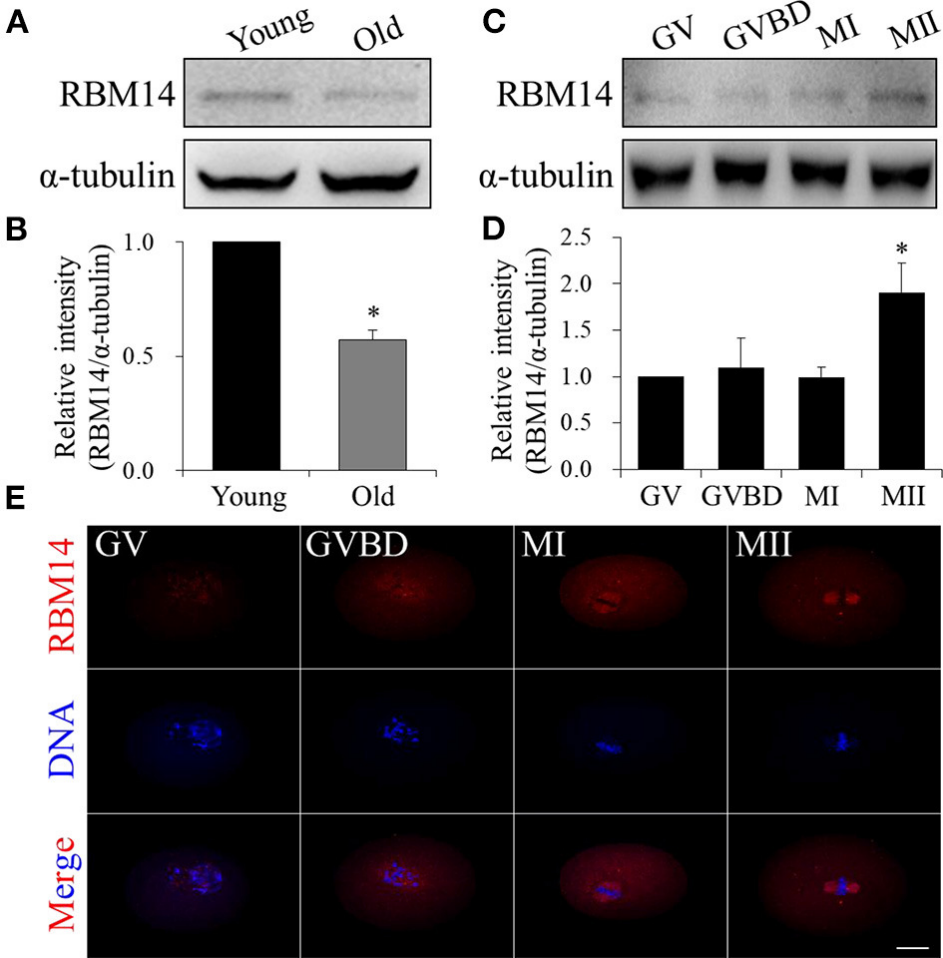
RBM14 expression in oocytes from aged mice and the expression and localization patterns of RBM14 during mouse oocyte maturation. **(A)** Western blot analysis showing RBM14 protein levels in oocytes from young (6–8 weeks) and old (42–45 weeks) mice. **(B)** Normalized band intensities: RBM14/α-tubulin. RBM14 protein levels were significantly lower in oocytes obtained from old mice compared to young mice. **(C)** RBM14 protein levels at GV (0 h), GVBD (2 h), MI (8 h), and MII (12 h) stages of young mice were detected by Western blot analysis; α-tubulin was the loading control. **(D)** Normalized band intensities: RBM14/α-tubulin. RBM14 protein levels were significantly higher in oocytes at MII stage compared to GV stage. ^*^P < 0.05. **(E)** Immunofluorescence staining: red = RBM14 antibody, blue = DNA (Hoechst 33342). Scale bar = 20 μm. Data are mean ± SEM of three independent experiments. ^*^P < 0.05.

Western blot analysis showed that RBM14 protein levels were significantly lower in oocytes obtained from mice aged 42–45 weeks (old) compared to mice aged 6–8 weeks (young) (normalized band intensities: 0.57 ± 0.04 vs. 1.0, *P* < 0.05; Figures 1A,B). During *in vitro* maturation, oocytes were collected at 0 (GV-stage), 2 (GVBD-stage), 8 (MI-stage) and 12 h (MII-stage). Immunoblotting verified that RBM14 protein levels were gradually increased from GV- to MII-stage oocytes (Figures 1C,D). These data imply that RBM14 is required for meiotic maturation and such a decrease could contribute to poor quality eggs that occur with age. Immunofluorescence staining revealed the subcellular localization of RBM14 protein during mouse oocyte meiotic maturation. RBM14 was concentrated in the nucleus in GV-stage oocytes, around the periphery of the chromosomes in GVBD-stage oocytes, and had a spindle-like pattern in MI and MII-stage oocytes (Figure 1E). The subcellular localization was confirmed using two different RBM14 antibodies (Supplementary Figure 1). Taken together, these results suggest that a scarcity of RBM14 protein may be associated with a decline in oocyte quality. Therefore, we speculate that there is a relationship between RBM14 and the spindle during mouse oocyte meiotic maturation.

### RBM14 Is Enriched in the Oocyte Spindle During Meiosis

**Figure 2:**
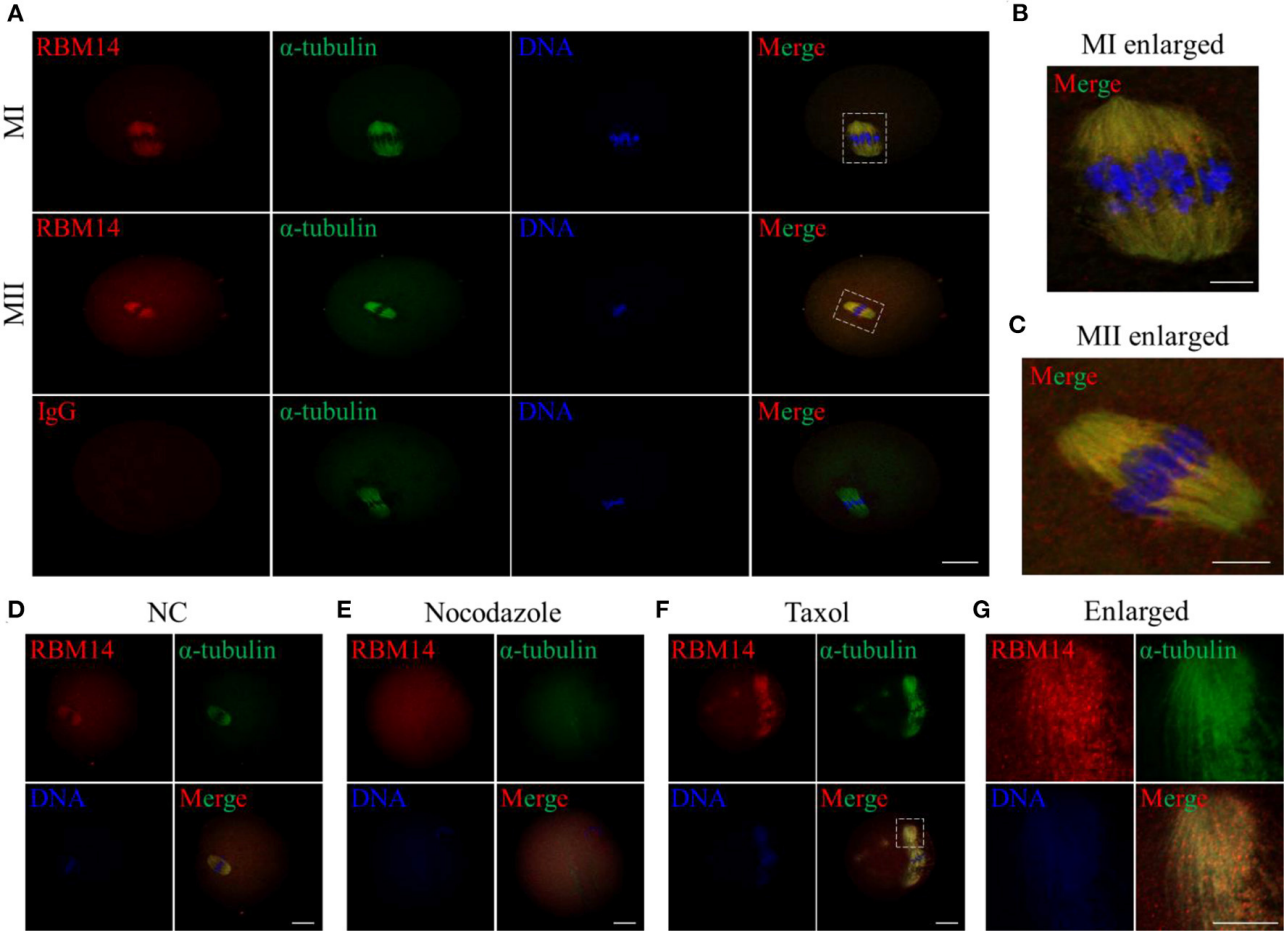
Localization of RBM14 in mouse oocytes treated with spindle-perturbing agents (nocodazole or taxol). **(A)** Immunofluorescence staining: red = RBM14 antibody, green = α-tubulin-FITC antibody, blue = DNA (Hoechst 33342). Representative images showing MI- and MII-stage oocytes. RBM14 signals were mainly distributed on spindle microtubules and presented a similar localization pattern to α-tubulin. The dotted boxes indicate the enlarged areas. No obvious signals were detected in the negative control (IgG antibody). Scale bar = 20 μm. **(B)** Magnified images of the white dotted box in **(A)**, MI-stage oocytes. Red = RBM14 antibody; green = α-tubulin-FITC antibody; blue = DNA (Hoechst 33342). Scale bar = 5 μm. **(C)** Magnified images of the white dotted box in **(A)**, MII-stage oocytes. Red = RBM14 antibody; green = α-tubulin-FITC antibody; blue = DNA. Scale bar = 5 μm. **(D–F)** MI-stage oocytes treated with nocodazole or taxol: red = RBM14 antibody, green = α-tubulin-FITC antibody, blue = DNA (Hoechst 33342). The dotted box indicates the enlarged area. Scale bars = 20 μm. **(G)** Magnified images of the white dotted box in **(F)**, MI-stage oocytes treated with taxol. Red = RBM14 antibody; green = α-tubulin-FITC antibody; blue = DNA (Hoechst 33342). Scale bar = 10 μm.

To provide further insight into the subcellular localization of RBM14, MI- and MII-stage oocytes were co-stained with RBM14 and α-tubulin-FITC antibodies. Of note, RBM14 was strongly distributed on spindle microtubules and presented overlapping localization patterns with α-tubulin in MI- and MII-stage oocytes (Figures 2A–C and Supplementary Figure 2); rabbit IgG was used as a negative control. Subsequently, we treated MI-stage oocytes with spindle-perturbing agents to verify whether RBM14 is co-localized with microtubules (Figure 2D, control group). After nocodazole treatment (20 μg/ml; 20 min), spindles were depolymerized and the RBM14 signal coincided with disassembled microtubules (Figure 2E). Next, MI-stage oocytes were exposed to taxol (10 μM; 40 min). Microtubule fibers were excessively polymerized and colocalized fluorescent signals of RBM14 and α-tubulin were observed at the cytoplasmic asters (Figures 2F,G). These findings indicate that the localization pattern of RBM14 was consistent with microtubules after treated with spindle-perturbing agents in mouse oocytes.

### Knockdown of RBM14 Affects Polar Body Extrusion and Asymmetric Division in Mouse Oocytes

**Figure 3:**
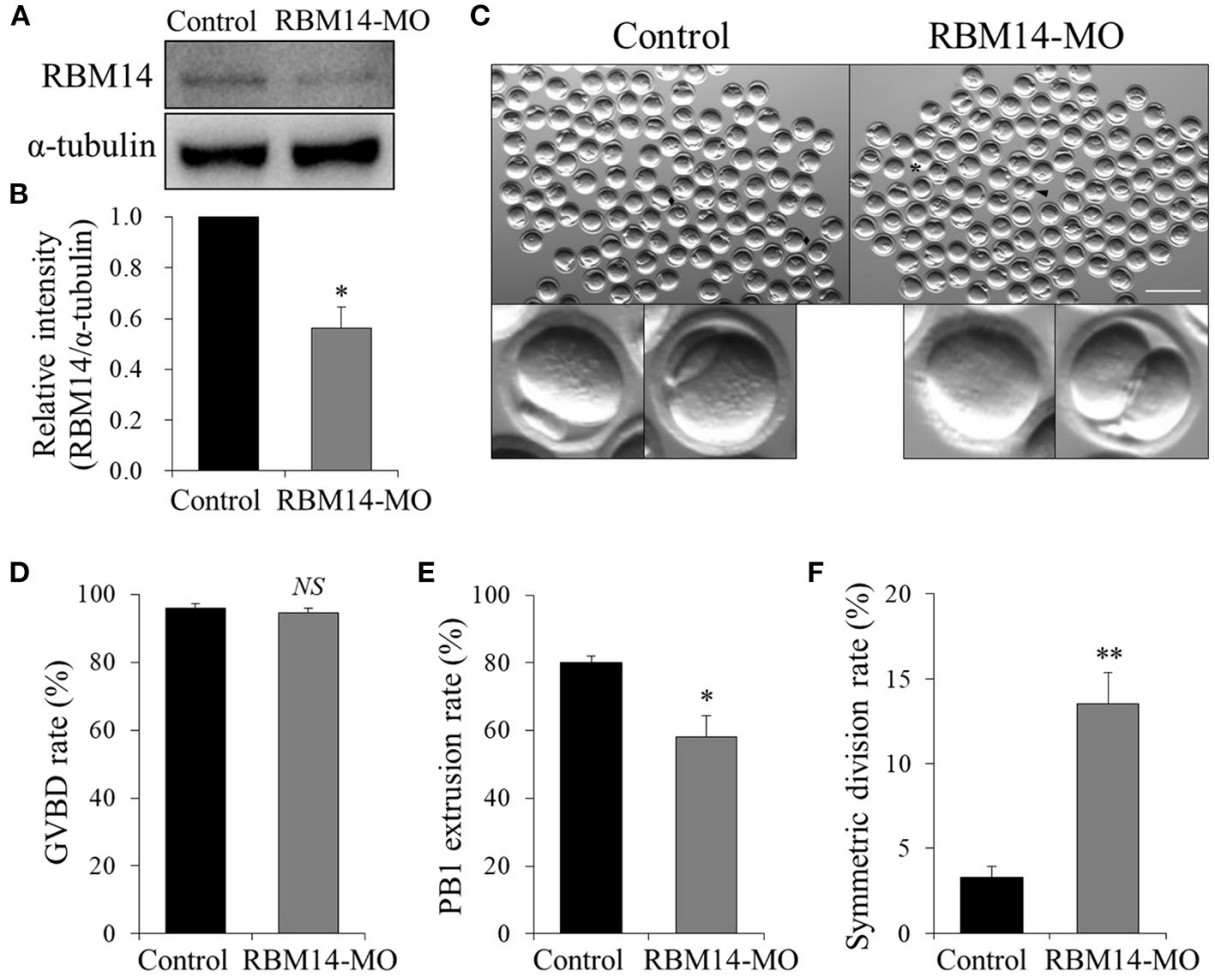
Effects of RBM14 MO on the maturation of mouse oocytes. **(A)** Western blot analysis verified the efficiency of endogenous RBM14 knockdown. **(B)** RBM14 band intensities normalized to α-tubulin. **(C)** Representative bright-field images of RBM14-MO injected and control oocytes. Black rhombuses: successful polar body extrusion in control oocytes; black arrow: oocyte with apparent symmetric division; black asterisk: oocyte failed to extrude a polar body. Scale bar = 200 μm. **(D)** Rate of GVBD in control and RBM14-MO injected oocytes. **(E)** Rate of polar body extrusion in control and RBM14-MO injected oocytes. **(F)** Rate of symmetric division in control and RBM14-MO injected oocytes. A total of 364 control oocytes and 332 RBM14-MO injected oocytes were analyzed. Data are mean ± SEM of three independent experiments. *NS*, not significant, *P < 0.05 and **P < 0.01.

To investigate the role of RBM14 during oocyte meiosis, we employed microinjection of RBM14 MO to deplete RBM14 protein in mouse oocytes. Western blot analysis revealed that RBM14 protein levels were significantly decreased in RBM14-MO injected oocytes compared to controls (0.56 ± 0.08 vs. 1.0, *P* < 0.05) (Figures 3A,B). Next, we explored the effect of RBM14 depletion on oocyte maturation. RBM14-MO injected oocytes were arrested at GV stage for 20 h in M2 medium supplemented with 2.5 μM milrinone and then cultured in fresh M16 medium. The proportion of GVBD was similar in RBM14-depleted oocytes compared to controls, indicating that reduced expression of RBM14 did not significantly affect meiotic resumption (94.58 ± 1.33% vs. 95.83 ± 1.46%, *P* > 0.05; Figure 3D). However, after culture for 12 h in M16 medium, there was a significant decrease in the first polar body (PB1) extrusion rate in RBM14-depleted oocytes compared to controls (58.17 ± 6.07% vs. 80.17 ± 1.80%, *P* < 0.05; Figure 3E), indicating that RBM14 plays an important role in the meiotic division and polar body formation (Figure 3C, black asterisk). Furthermore, RBM14-depleted oocytes underwent symmetric division compared to controls (Figure 3C, black arrowhead; 13.51 ± 1.87% vs. 3.31 ± 0.64%, *P* < 0.01; Figure 3F). These results suggest that RBM14 has a critical role in mouse oocyte maturation.

### Knockdown of RBM14 in Mouse Oocytes Affects Normal Spindle Morphology and Chromosome Alignment

**Figure 4:**
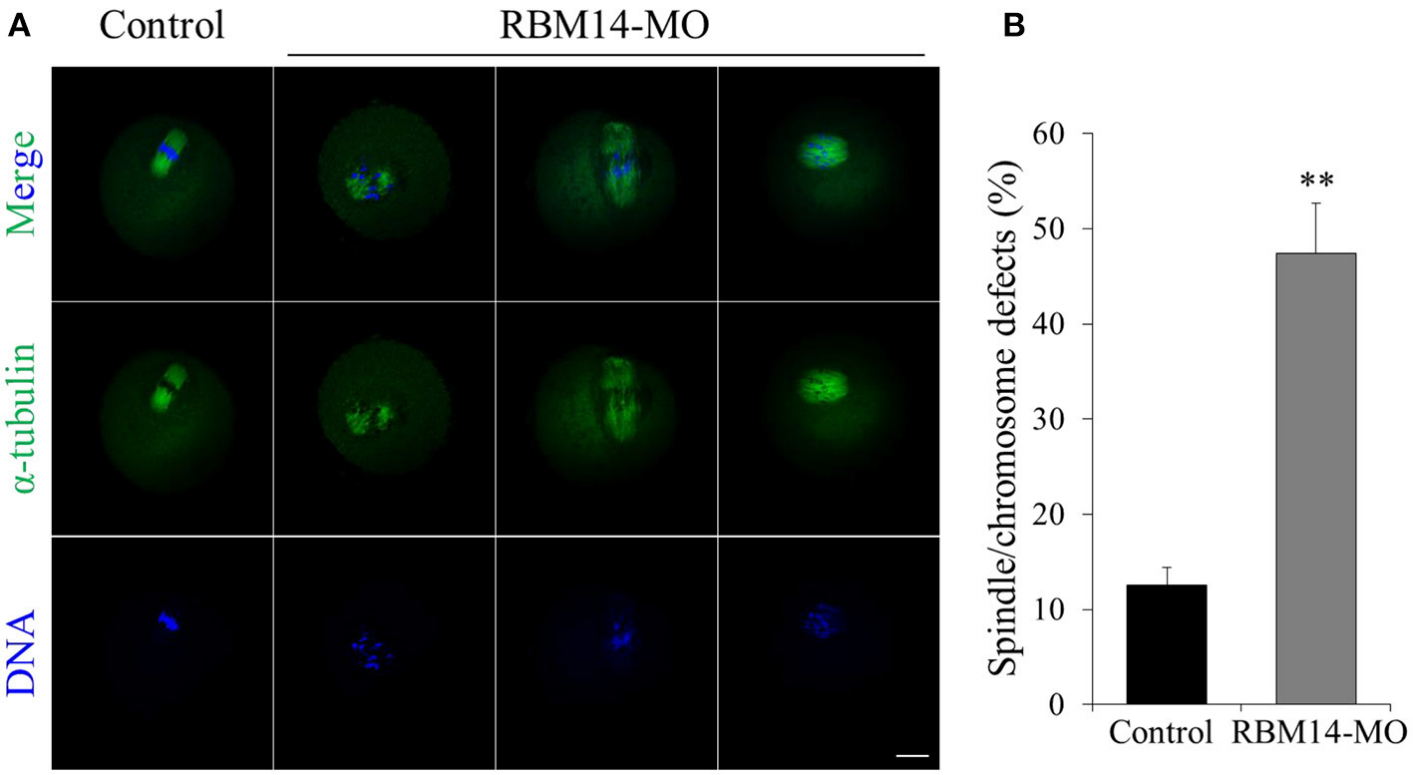
RBM14 depletion leads to abnormal spindle morphology and chromosome misalignment in mouse oocytes. **(A)** Confocal images of control and RBM14-depleted oocytes. Green = α-tubulin-FITC antibody, blue = DNA (Hoechst 33342). In control oocytes, meiotic spindles presented normal barrel-shaped morphologies and well-aligned chromosomes. In RBM14-MO injected oocytes, meiotic spindles presented abnormal morphology (e.g., apolar and malformed) and irregularly scattered chromosomes. Scale bar = 20 μm. **(B)** Percentage of oocytes with spindle/chromosome defects. Data are mean ± SEM of three independent experiments. **P < 0.01.

The above data prompted us to investigate the reasons for the oocyte maturation defects caused by RBM14 deficiency. RBM14 knockdown oocytes were developed to MI stage after 8 h culture and co-stained with anti-α-tubulin-FITC antibody and Hoechst 33342 to observe spindle morphology and chromosome alignment. The meiotic spindles of the control group presented normal barrel-shaped morphologies and well-aligned chromosomes. In marked contrast, RBM14-MO injected oocytes exhibited abnormal spindles, including apolar and malformed spindles and irregularly scattered chromosomes (12.57 ± 1.88%; *n* = 62 vs. 47.45 ± 5.21%; *n* = 66, *P* < 0.01; Figures 4A,B). These results suggest that RBM14 is essential for normal spindle morphology and chromosome alignment.

### Depletion of RBM14 Increases Acetylation of α-Tubulin in Mouse Oocytes and RBM14 Interacts With α-Tubulin in Mammalian Cells

**Figure 5:**
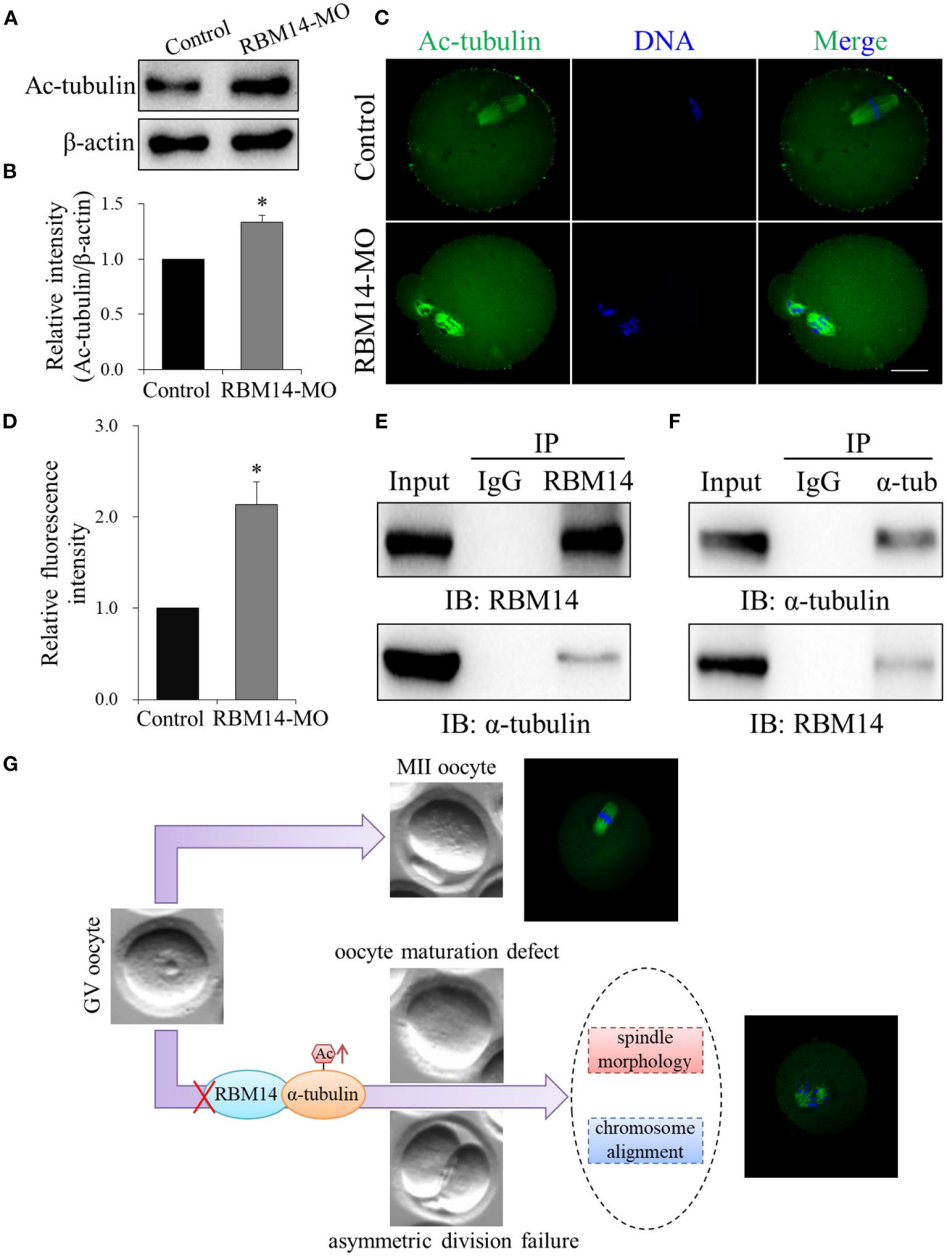
RBM14 depletion increased α-tubulin acetylation in mouse oocytes by interacting with α-tubulin. **(A)** Western blot analysis showing that the level of α-tubulin acetylation was increased in RBM14-MO injected oocytes. **(B)** Acetylated α-tubulin band intensities normalized to β-actin. **(C)** Representative confocal images of control and RBM14-depleted oocytes. Green = ac-tubulin antibody, blue = DNA (Hoechst 33342). Scale bar = 20 μm. **(D)** Relative fluorescence intensities of acetylated α-tubulin in control and RBM14-depleted oocytes. **(E)** Co-IP was performed with whole cell extracts from NIH/3T3 cells to determine the interaction between RBM14 and α-tubulin. Protein lysate was incubated with RBM14 antibody or nonspecific rabbit IgG antibody, followed by Western blot analysis using an α-tubulin antibody. **(F)** Reciprocal co-IP in NIH/3T3 whole cell lysates using α-tubulin antibody or nonspecific rabbit IgG antibody to pull down α-tubulin, followed by Western blot analysis using a RBM14 antibody. **(G)** A schematic model showing the potential pathway that RBM14 is involved in oocyte meiotic maturation. Data are mean ± SEM of three independent experiments. *P < 0.05.

A previous study demonstrated that the acetylation of α-tubulin was essential for microtubule structure and stability (Eshun-Wilson et al., 2019). Therefore, we investigated whether RBM14 modulates α-tubulin acetylation during meiosis in oocytes. Western blot analysis (1.33 ± 0.06 vs. 1.0, *P* < 0.05; Figures 5A,B) and immunofluorescence (2.14 ± 0.25; *n* = 55 vs. 1.0; *n* = 40, *P* < 0.05; Figures 5C,D) revealed that the level of α-tubulin acetylation was significantly higher in RBM14-MO injected oocytes compared to controls. These data imply that RBM14 regulates the acetylation of α-tubulin to modulate microtubule stability. The increased level of α-tubulin acetylation in RBM14-depleted oocytes prompted us to explore the relationship between RBM14 and α-tubulin. We performed the co-IP experiment with RBM14 or α-tubulin antibodies separately using NIH/3T3 whole cell lysates. Endogenous RBM14 was pulled down by RBM14 antibody and then the blot of the IP eluate was probed with an α-tubulin antibody. As shown in Figure 5E, α-tubulin was specifically precipitated with the RBM14 antibody, and rabbit IgG served as the negative control. Reciprocally, immunoblotting confirmed that RBM14 was detected in α-tubulin immunoprecipitated fraction (Figure 5F). These results suggest a physical interaction between RBM14 and α-tubulin.

## Discussion

Mammalian oocyte maturation is a precisely regulated process. The day before ovulation, immature GV-stage oocytes complete meiosis I, forming the first polar body and a haploid egg. The first polar body is extruded and the egg commences meiosis II, which arrests at metaphase until fertilization. The mechanisms controlling the formation of the meiotic spindle in mammalian oocytes remain to be elucidated. The results of the present study showed that RBM14 protein was gradually increased from GV- to MII-stage mouse oocytes and RBM14 depletion caused spindle abnormalities and deficiencies in chromosome segregation. These findings prompted us to ask whether RBM14 localization correlates with microtubule. The use of spindle perturbing agents revealed that RBM14 consistently localized to spindle microtubules, with localization in proximity to the oocyte chromosomes dependent on microtubules, while depletion of RBM14 protein in oocytes with morpholino resulted in abnormal polar body extrusion and symmetric meiotic division during mouse oocyte maturation. In accordance with these findings, previous reports demonstrated that the RBM gene family actively participates in the biological processes of reproduction and embryogenesis. Specifically, in human cells, RBM14 preserved mitotic spindle integrity by suppressing the formation of aberrant centriolar protein complexes in the cytoplasm and regulating centriole biogenesis (Shiratsuchi et al., 2015). In the mouse, a homolog of the human Y-linked *RBM* genes was expressed in the testis and presented a maximal amount at 14 days postpartum, which corresponded to the time that the Y chromosome condensed during meiotic prophase (Elliott et al., 1996). Furthermore, RBM10 knockout led to a marked decrease in the proliferation of mouse embryonic stem cells and dramatically affected the size of embryoid bodies (Rodor et al., 2017). Similarly, RBM14 maintained genome integrity during early mouse embryogenesis and was indispensable for the pluripotency of mouse embryonic stem cells and mesoderm development (Chen et al., 2018; Li et al., 2020). In this study, we show that RBM14 is co-localized with the spindle during meiotic progression in the mouse oocytes. To investigate the functional involvement of RBM14, we employed specific morpholino injection to deplete RBM14 protein that resulted in abnormal polar body extrusion and symmetric meiotic division during mouse oocyte maturation.

Microtubule-based functions are affected by a variety of post-translational modifications, including acetylation, tyrosination, detyrosination, polyglutamylation, and polyglycylation (Fukushima et al., 2009). Acetylation of tubulin subunits can affect the structure and stability of microtubules, and molecular dynamics simulations showed that acetylation of α-tubulin on K40, which is localized to the inside of the microtubule, restricted the range of motion of the αK40 loop (Eshun-Wilson et al., 2019). In mammalian oocytes, the abnormal acetylation status of α-tubulin caused spindle disruption and chromosome misalignment. For example, in mouse oocytes, depletion of Sirt2 resulted in spindle defects and chromosome disorganization by increasing α-tubulin acetylation during meiosis (Zhang et al., 2014). Recently, Tang et al. reported that abnormal α-tubulin K40 acetylation level mediated by Kif4a led to spindle organization defects and chromosome alignment failure in mouse oocytes (Tang et al., 2018a). In the present study, RBM14 depletion induced spindle defects and chromosome misalignment caused by excessive accumulation of acetylated α-tubulin, representing a potential molecular mechanism involved in the decline of oocyte quality.

RBM14 belongs to the family of RNA-binding proteins and harbors two highly conserved RRMs at the N-terminus and an unstructured prion-like domain at the C-terminus. Previous studies have revealed that RBM14 may affect various cellular processes through protein-protein physical interactions. *In vitro* and *in vivo*, RBM14 interacted with the proto-oncoprotein SYT to stimulate hormone receptor-dependent transcriptional activation (Perani et al., 2005). *In vitro*, RBM14 directly interacted with thyroid hormone receptor-binding protein and p300 to modulate gene activation (Iwasaki et al., 2001). In line with this conception, our data showed that RBM14 interacted with α-tubulin and might be required for its acetylation. This observation is similar to a previous study that showed the RNA-binding protein EWSR1 interacts with α-tubulin and regulates the acetylation of microtubules during mitosis (Wang et al., 2016). At the same time, we wanted to elucidate the potential molecular mechanism underlying how RBM14 modulates α-tubulin acetylation. Intriguingly, the modulation of acetylation by RBM14 on its binding protein fits well with a previous report showing Hu RNA-binding proteins directly interact with and inhibit the deacetylation activity of histone deacetylase 2 (Zhou et al., 2011). Furthermore, in fission yeast, a homolog of human splicing factor RBM10 was associated with the histone deacetylase Clr6 complex and facilitated heterochromatin assembly (Weigt et al., 2020). Based on these data, we speculate that RBM14 might interact with the histone deacetylase family proteins, such as HDAC3 or Sirt2, which has been reported to regulate microtubule stability and spindle organization through deacetylating α-tubulin (Li et al., 2017; Tang et al., 2018b). Hence, we cannot rule out the possibility that RBM14 could modulate histone deacetylase activities and form a tripartite protein complex with α-tubulin to co-regulate the acetylation of α-tubulin. In conclusion, this study indicates that RBM14 is an essential modulator of oocyte meiotic maturation by regulating α-tubulin acetylation to affect spindle morphology and chromosome alignment (Figure 5G). Consequently, RBM14 represents a potential biomarker of oocyte quality and a novel therapeutic target in women with oocyte maturation failure.

## Data Availability Statement

The raw data supporting the conclusions of this article will be made available by the authors, without undue reservation.

## Ethics Statement

The animal study was reviewed and approved by the Institutional Animal Care and Use Committee of Peking University.

## Author Contributions

JQ designed and supervised the project. HQ and YQ performed the experiments, collected, and analyzed the data. HQ and JQ wrote the manuscript and revised it. JQ, HQ, and YQ provided the funding. Y-FY and Y-YL provided the technical support. All authors contributed to the article and approved the submitted version.

## Conflict of Interest

The authors declare that the research was conducted in the absence of any commercial or financial relationships that could be construed as a potential conflict of interest.

## Supplementary Material

Supplementary Figure 1 Confirmation of the subcellular localization of RBM14 in mouse oocytes. Representative images of oocytes during meiosis stained with anti-RBM14 antibody (red, ab70636) and counterstained with Hoechst 33342 for DNA (blue). Scale bar = 20 μm.

Supplementary Figure 2 Localization of RBM14 to meiotic spindles in mouse oocytes. Immunofluorescence staining with anti-RBM14 antibody (red, ab70636) colocalized with α-tubulin (green) in MII-stage oocytes. Blue = DNA (Hoechst 33342). Scale bars = 20 μm.
