# Supplementary figures and images for: RBM14 Modulates Tubulin Acetylation and Regulates Spindle Morphology During Meiotic Maturation in Mouse Oocytes
Source: Front Cell Dev Biol. 2021 Feb 2;9:635728. doi: 10.3389/fcell.2021.635728 (PMC7884444; doi:10.3389/fcell.2021.635728)

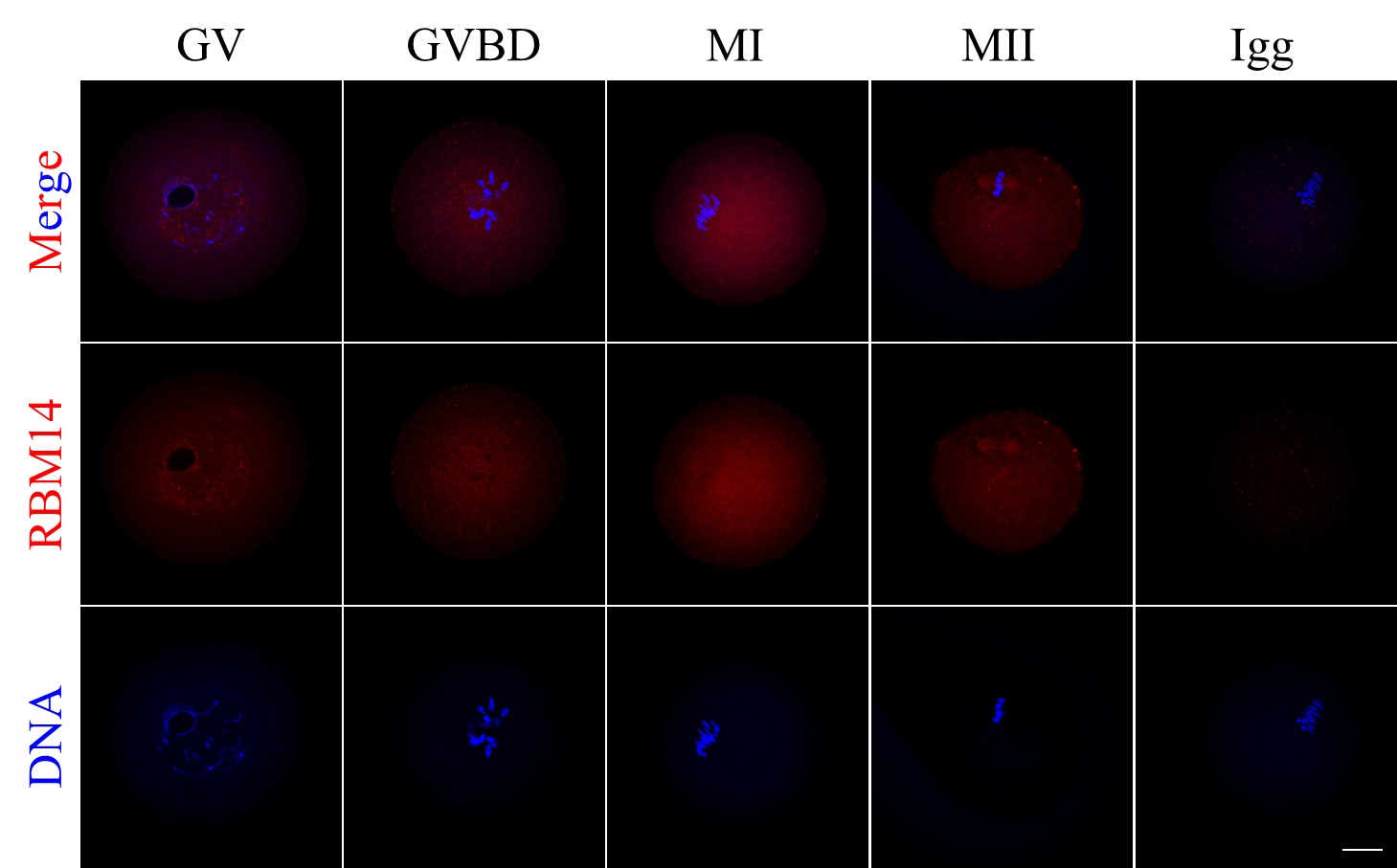

Supplement: Supplementary Figure 1 — Confirmation of the subcellular localization of RBM14 in mouse oocytes. Representative images of oocytes during meiosis stained with anti-RBM14 antibody (red, ab70636) and counterstained with Hoechst 33342 for DNA (blue). Scale bar = 20 μm. [file Image_1.TIF]

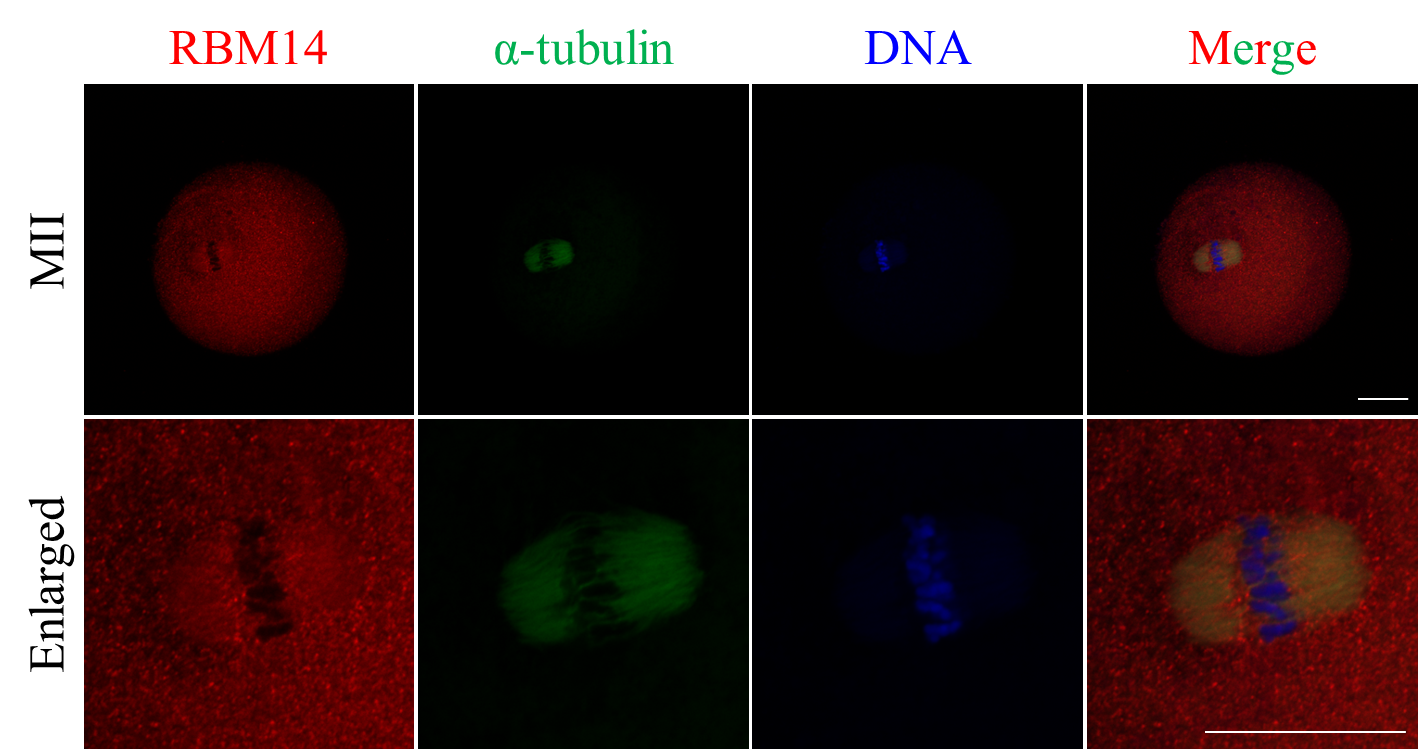

Supplement: Supplementary Figure 2 — Localization of RBM14 to meiotic spindles in mouse oocytes. Immunofluorescence staining with anti-RBM14 antibody (red, ab70636) colocalized with α-tubulin (green) in MII-stage oocytes. Blue = DNA (Hoechst 33342). Scale bars = 20 μm. [file Image_2.TIF]
